# Established and emerging therapeutic uses of PDE type 5 inhibitors in cardiovascular disease

**DOI:** 10.1111/bph.14920

**Published:** 2020-02-04

**Authors:** Nikolaos Tzoumas, Tariq E. Farrah, Neeraj Dhaun, David J. Webb

**Affiliations:** ^1^ British Heart Foundation/University Centre for Cardiovascular Science, Queen's Medical Research Institute University of Edinburgh Edinburgh UK; ^2^ Institute of Genetic Medicine Newcastle University Newcastle Upon Tyne UK

## Abstract

PDE type 5 inhibitors (PDE5Is), such as sildenafil, tadalafil and vardenafil, are a class of drugs used to prolong the physiological effects of NO/cGMP signalling in tissues through the inhibition of cGMP degradation. Although these agents were originally developed for the treatment of hypertension and angina, unanticipated side effects led to advances in the treatment of erectile dysfunction and, later, pulmonary arterial hypertension. In the last decade, accumulating evidence suggests that PDE5Is may confer a wider range of clinical benefits than was previously recognised. This has led to a broader interest in the cardiovascular therapeutic potential of PDE5Is, in conditions such as hypertension, myocardial infarction, stroke, peripheral arterial disease, chronic kidney disease and diabetes mellitus. Here, we review the pharmacological properties and established licensed uses of this class of drug, along with emerging therapeutic developments and possible future indications.

**Abbreviations**
6MWD: 6‐min walking distance
ACEI: ACE inhibitor
ARB: angiotensin receptor blocker
BPS: British Pharmacological Society
CCB: calcium channel blocker
CKD: chronic kidney disease
*C*_max_: maximum plasma/serum concentration
CTEPH: chronic thromboembolic pulmonary hypertension
CVD: cardiovascular disease
CYP2C19: cytochrome P450, family 2, subfamily C, polypeptide 19
CYP2C9: cytochrome P450, family 2, subfamily C, polypeptide 9
CYP2D6: cytochrome P450, family 2, subfamily D, polypeptide 6
CYP3A: cytochrome P450, family 3, subfamily A
CYP3A4: cytochrome P450, family 3, subfamily A, polypeptide 4
CYP450: cytochrome P450 enzyme family
DN: diabetic nephropathy
ED: erectile dysfunction
eGFR: estimated GFR
ERA: endothelin receptor antagonist
ET: endothelin
ET‐1: endothelin‐1
FGR: fetal growth restriction
GSK3B: glycogen synthase kinase 3 β
HbA1c: glycated Hb
HFpEF: heart failure with preserved ejection fraction
HFrEF: heart failure with reduced ejection fraction
IC_50_: half maximal inhibitory concentration
IUPHAR: International Union of Basic and Clinical Pharmacology
MI: myocardial infarction
mitoK_ATP_: mitochondrial ATP‐sensitive potassium channels
NAION: nonarteritic anterior ischaemic optic neuropathy
Na^+^/K^+^‐ATPase: sodium–potassium pump
NOS1: neuronal NOS
NOS2: inducible NOS
NOS3: endothelial NOS
PAH: pulmonary arterial hypertension
PDE5I: PDE type 5 inhibitor
PH: pulmonary hypertension
PPHN: persistent pulmonary hypertension of the newborn
RCT: randomised controlled trial
RHTN: treatment‐resistant hypertension
RP: Raynaud's phenomenon
sGC: soluble GC
SGLT2: sodium–glucose cotransporter 2
*T*_max_: time taken to reach the maximum plasma concentration
T2DM: type 2 diabetes mellitus
UACR: urinary albumin/creatinine ratio
V/Q: ventilation/perfusion

## 1 INTRODUCTION

**FIGURE 1:**
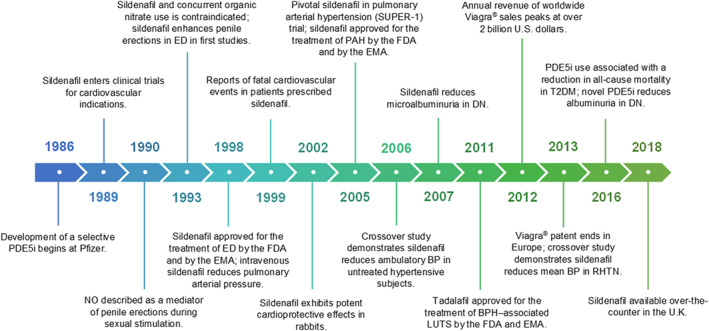
Milestones in the development of PDE type 5 inhibitors (PDE5Is). The figure depicts the key milestones in the development of PDE5Is for clinical indications, from their inception and early clinical trials, to their success in the amelioration of several cardiovascular conditions and finally their availability as an over‐the‐counter medication. BP, blood pressure; BPH, benign prostatic hyperplasia; DN, diabetic nephropathy; ED, erectile dysfunction; EMA, European Medicines Agency; FDA, U.S. Food and Drug Administration; LUTS, lower urinary tract symptoms; NO, nitric oxide; PAH, pulmonary arterial hypertension; PDE5I, PDE type 5 inhibitor; RHTN, treatment‐resistant hypertension; T2DM, type 2 diabetes mellitus

Few other pharmaceutical agents have had a history as serendipitous as the class of PDE type 5 inhibitors (PDE5Is; Figure 1). Developed over 30 years ago, initially for cardiovascular indications, PDE5Is emerged as a revolutionary treatment for erectile dysfunction (ED), licensed in 1998 (Goldstein, Lue et al., 1998). Further investigation of their effects on pulmonary arterial pressure culminated in their approval for the treatment of pulmonary arterial hypertension (PAH) in 2005 (Galiè, Ghofrani et al., 2005). Early reports of fatal cardiovascular events in patients prescribed a PDE5I delayed wider research into their therapeutic benefits, but it soon became apparent that these adverse events reflected the complex nature of patients initially receiving the drug and their high level of cardiovascular risk, rather than the properties of the drug itself (Cheitlin, Hutter et al., 1999; Giuliano, Jackson et al., 2010). With a growing body of evidence supporting the clinically established safety profile and efficacy of these agents, PDE5Is became available as over‐the‐counter pharmacy medicines in the United Kingdom in early 2018. Now, there is renewed research interest in their therapeutic potential in conditions including cardiovascular disease (CVD), chronic kidney disease (CKD) and diabetes mellitus. This narrative literature review discusses the historical and biochemical basis for the established uses of PDE5Is and presents emerging evidence of their utility in novel indications.

## 2 METHODS

We conducted a literature search of multiple electronic databases including Medline, Embase, Cochrane Library, Google Scholar and Trip medical database over the period of December 2016 to September 2019. Search terms included, but were not limited to, phosphodiesterase type 5, phosphodiesterase type 5 inhibitors, erectile dysfunction, pulmonary hypertension, lower urinary tract symptoms, cardiovascular disease, blood pressure, stroke, connective tissue disease, chronic kidney disease and diabetes mellitus. One hundred and fifty articles were included in our qualitative synthesis and are referred to in this review.

## 3 BIOCHEMISTRY

Before discussing the current use of PDE5Is and their new clinical indications, we briefly review the underlying cellular and molecular processes. Where relevant, endogenous ligands and drugs mentioned in the text are hyperlinked directly to The International Union of Basic and Clinical Pharmacology (IUPHAR) /British Pharmacological Society (BPS) Guide to Pharmacology (https://www.guidetopharmacology.org), an online, expert‐curated database with up‐to‐date information on drug targets and the substances that act on them (Harding, Sharman et al., 2018).

**FIGURE 2:**
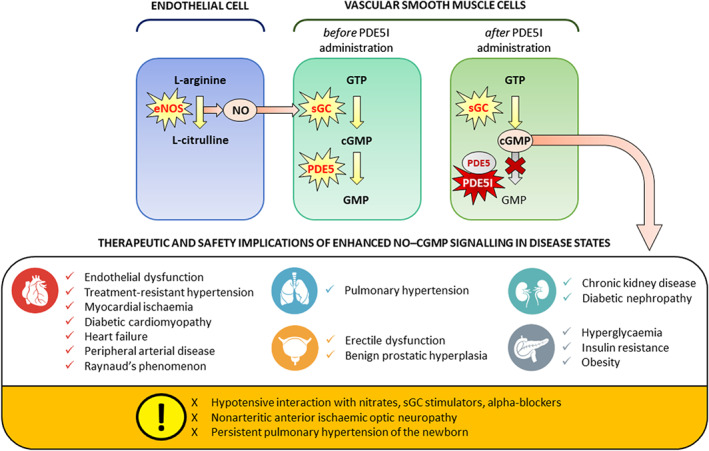
The NO–cGMP signalling pathway in the vasculature. The figure shows the generator cells, for example, vascular endothelial cells, the target cells, for example, vascular smooth muscle cells and biochemical processes involved in NO signalling in the vasculature. NO is produced through the conversion of the substrate l‐arginine to l‐citrulline by endothelial NO synthase (NOS3) in vascular endothelial cells. Subsequently, NO diffuses to neighbouring vascular smooth muscle cells where it activates soluble GC (sGC), which converts guanosine triphosphate (GTP) to cGMP. cGMP is a secondary messenger enacting cellular processes through the regulation of protein‐dependent kinases, for example, PKG, and cGMP‐gated ion channels. The cellular actions of cGMP can be prolonged by PDE type 5 inhibitors (PDE5Is), which prevent its degradation by PDE type 5 (PDE5), resulting in vasodilatory, antioxidative and anti‐proliferative effects in several organ systems and diseases. Icons with permission from ^©^ iStock.com/Alex Doubovitsky

Cyclic nucleotide PDEs are a diverse family of enzymes that regulate the cellular levels of the secondary messenger molecules cAMP and cGMP by catalysing the degradation of their phosphodiester bond (Boswell‐Smith, Spina, & Page, 2006). The superfamily of PDE enzymes is classified into 11 major isozyme groups, namely, PDE1–PDE11, each with different substrate specificities: PDE5 together with PDE6 and PDE9 are selective cGMP hydrolases, whereas other PDEs are cAMP‐selective (PDE4, PDE7 and PDE8) or have mixed specificity (PDE1, PDE2, PDE3, PDE10 and PDE11; Boswell‐Smith, Spina, & Page, 2006). Although PDE5 is abundantly distributed in vascular smooth muscle, PDE5 mRNA and isoform expression has also been identified in various human organs, including brain, lung, heart, liver, kidney, bladder, prostate, urethra, penis, uterus and skeletal muscle, where it performs numerous physiological functions mediated by the NO–cGMP pathway (Figure 2; Boswell‐Smith, Spina, & Page, 2006). Due to its widespread tissue distribution and functions, PDE5 has been established as an attractive target for pharmacological intervention to prolong or enhance the effects of processes mediated by cGMP by inhibiting its degradation (Boswell‐Smith, Spina, & Page, 2006).

cAMP and cGMP play key roles in cell signalling through the activation of intracellular protein kinases which modulate several metabolic processes such as ion conductance, cellular apoptosis and glycogenolysis. In smooth muscle cells, for example, the binding of cGMP to the cGMP‐dependent PKG causes a reduction in intracellular calcium concentrations and, subsequently, relaxation of the smooth muscle layer and vasodilatation (Boswell‐Smith, Spina, & Page, 2006). cGMP is synthesised from GTP by soluble GC (sGC), a heterodimeric enzyme which is itself activated by NO. NO is a cellular signalling molecule implicated in a wide range of regulatory functions, including control of vascular tone, platelet aggregation and cell communication, as well as in the immunological response to infection, chronic inflammation and tumours (Ignarro, 2019). NO is synthesised endogenously from the oxidation of the amino acid l ‐arginine to l ‐citrulline under the effect of NOS, of which there are three isoforms, neuronal NOS (NOS1), inducible NOS (NOS2) and endothelial NOS (NOS3), but can also be derived from the reduction of exogenously administered nitrates (Stuehr & Haque, 2019).

Vascular smooth muscle relaxation is also mediated by the effects of the prostacyclin pathway, whereby arachidonic acid is cleaved by cyclooxygenase to synthesise prostacyclin, a lipid molecule with potent vasodilatory, anti‐proliferative and anti‐platelet effects mediated through interactions with cell surface and nuclear receptors, which ultimately result in increased cAMP production and PKA activation (Mitchell & Kirkby, 2019). Local vascular tone is regulated by an interaction between these relaxing processes and the effects of constrictor actions, including the endothelin (ET) pathway, primarily mediated by secretion of the potent vasoconstrictor endothelin‐1 (ET‐1) from vascular endothelial cells. Cumulative evidence suggests that the ET pathway opposes the NO–cGMP pathway and contributes to the pathogenesis of several cardiovascular and inflammatory diseases. Pharmacological manipulation of this system via inhibition of ET biosynthesis or direct antagonism of ET receptors has generated significant experimental interest and potential therapeutic applications (Dhaun & Webb, 2019).

The vasodilatory properties of the NO–cGMP pathway have been successfully exploited for the management of ischaemic heart disease, whereby exogenous nitrate administration in the form of nitroglycerin reduces peripheral vascular resistance and cardiac preload to improve myocardial perfusion. However, despite its potent anti‐anginal effect, such practice is limited by the rapid development of nitrate tolerance with sustained exogenous administration. In an attempt to circumvent this issue, research interest in the mid‐1980s shifted to the modulation of intracellular cGMP levels and its downstream effectors (Beavo & Reifsnyder, 1990).

## 4 DEVELOPMENT OF PDE TYPE 5 INHIBITORS

In 1986, Pfizer began preclinical work on the development of a selective PDE5‐inhibitor (PDE5I) to compete with cGMP for access to the PDE5 catalytic site. It was proposed that such an agent could prolong the effects of NO–cGMP signalling in vascular tissue and thus ameliorate conditions associated with NO deficiency, such as angina pectoris. In preclinical studies, the resulting agent — later named sildenafil citrate — demonstrated potent vasodilatory effects and was selected for further development (Terrett, Bell et al., 1996). In 1991, sildenafil entered clinical trials for use in cardiovascular indications. In single‐dose trials, the agent produced reductions in systemic BP in healthy volunteers, as well as modest vasodilatory and anti‐platelet effects when administered intravenously to patients with ischaemic heart disease (Boswell‐Smith, Spina, & Page, 2006; Jackson, Benjamin et al., 1999). Despite these initial promising results, research on the cardiovascular benefits of PDE5I therapy was complicated by the short plasma half‐life of sildenafil of around 4 hr, which would necessitate frequent dosing for the chronic treatment of angina. Development was further compounded by the contraindication of concurrent nitrate use within 24 hr of PDE5I ingestion (48 hr for tadalafil) due to the risk of a significant synergistic vasodilatory interaction between the agents on the systemic vasculature, leading to dangerous levels of hypotension in recipients (Webb, Freestone et al., 1999).

## 5 THERAPEUTIC APPLICATION OF PDE TYPE 5 INHIBITORS

### 5.1 Erectile dysfunction (ED)

As multiple‐dose Phase I trials of sildenafil for the treatment of angina continued, healthy volunteers began reporting an unexpected and, in hindsight, fortuitous side effect: penile erections. ED, defined as the inability to attain or maintain a penile erection sufficient for successful vaginal intercourse (Lue, 2000), had, until the 1990s, been a clinically neglected issue, despite its global burden. At the time, therapeutic options for ED were limited to experimental intracavernosal injections of prostacyclin analogues or unlicensed antidepressants and herbal remedies, which had limited effectiveness and potential harms (Goldstein, Lue et al., 1998). A breakthrough in our understanding of this condition came in 1990, when NO was identified as a key neurotransmitter facilitating penile erections. Its release into the penile vasculature at high levels from the endothelium and parasympathetic cavernous nerve terminals during sexual stimulation led to cGMP‐mediated relaxation of corpus cavernosal smooth muscle with subsequent vasocongestion, occlusion of local venous return, and a sustained erection (Ignarro, Bush et al., 1990). By 1993, the future of sildenafil as a treatment for angina looked increasingly uncertain. However, the occurrence of penile erections, linked with a clear mechanistic underpinning, provided the stimulus for the first clinical trials of PDE5Is for the treatment of ED.

The results from the first clinical study investigating PDE5I treatment in ED were unequivocally positive: Through the inhibition of cGMP degradation in the cavernous smooth muscles, sildenafil enhanced and prolonged the vasodilatory response of cGMP within the corpora cavernosa during sexual stimulation in a dose‐dependent manner to facilitate penile erection, without any safety concerns (Boolell, Allen et al., 1996). Later trials would confirm the high response rates, with the benefits of sildenafil treatment extending to ED associated with CVD, neurological disease, diabetes mellitus and radical prostatectomy (Lue, 2000).

In March 1998, sildenafil citrate (Viagra®; Pfizer; New York City, New York, USA) was approved for use in both the United States and the European Union as the first‐line treatment for ED secondary to a broad variety of physical causes (Goldstein, Lue et al., 1998; Ignarro, 2019); within 8 months, more than six million prescriptions of sildenafil were written in an unparalleled maelstrom of publicity (Lue, 2000). The testing and approval of other PDE5Is for the treatment of ED soon followed, culminating in 2003 with U.S. Food and Drug Administration and European Medicines Agency approval of tadalafil (Cialis®; Eli Lilly and Company; Indianapolis, Indiana, USA), the longest acting PDE5I,and vardenafil (Levitra®; Bayer; West Haven, Connecticut, USA), the first PDE5I to be available in both film‐coated and oro‐dispersible tablet forms to facilitate adherence in difficult patient groups. This was followed in 2012 by the introduction of avanafil (Stendra®; Vivus, Incorporated; Mountain View, California, USA), a more PDE5‐selective agent with favourable tolerability. Alternative PDE5Is have also been licensed in some countries: udenafil is a rapid‐onset but long‐acting agent available in South Korea, Russia and the Philippines (Paick, Kim et al., 2008); mirodenafil is a short‐acting PDE5I which has been extensively studied in ED but is currently only marketed in South Korea (Paick, Ahn et al., 2008); lodenafil is another short‐acting agent, which is only available in Brazil (Glina, Toscano et al., 2009). The clinical implications of these pharmacokinetic and pharmacodynamic variations in PDE5Is are explored further in the pharmacology and safety section.

### 5.2 Pulmonary hypertension

The demonstration of safe, consistent vasodilatation by sildenafil during treatment for ED generated renewed interest in the potential effects of PDE5 inhibition in other vascular beds. Pulmonary hypertension (PH) is a disorder characterised by a progressive increase in pulmonary vascular resistance resulting in right heart failure. Five subclasses of PH have been defined (as per the classification of the 6th World Symposium on Pulmonary Hypertension Task Force), among which Group 1 represents PAH, a term which encompasses a number of heterogeneous conditions sharing common pathophysiological, histological and prognostic features (Simonneau, Montani et al., 2019). Prior to 2005, treatment options for PAH included prostacyclin and prostacyclin analogue therapy, and the non‐selective ET receptor antagonist (ERA) bosentan. Although these drugs were successful in improving prognosis and exercise capacity in individuals with PAH, they were limited by concerns over safety, tolerability and ease of delivery (Simonneau, Montani et al., 2019). However, it should be noted that these issues have since been addressed following the development of novel ERAs. Both ambrisentan, an ET‐A selective agent, and macitentan, a dual ERA with a long half‐life and sustained receptor binding, have demonstrated favourable efficacy and tolerability profiles in the treatment of PAH (Galiè, Olschewski et al., 2008; Pulido, Adzerikho et al., 2013).

Local NO production regulates ventilation/perfusion (V/Q) matching as its production is up‐regulated in response to alveolar distension, leading to redirection of blood flow to well‐ventilated areas of the lung (Ghofrani, Pepke‐Zaba et al., 2004). PDE5 is highly expressed in healthy lung tissue (Corbin, Beasley et al., 2005) and up‐regulation of PDE5 gene expression was first observed in the lungs of rats with PH (Sanchez, De La Monte et al., 1998). Moreover, the weak and relatively non‐selective PDE5Is zaprinast and dipyridamole improved pulmonary pressure in models of PH (Ichinose, Adrie et al., 1998; Ziegler, Ivy et al., 1998). These factors, combined with the prospect of a well‐tolerated oral agent to enhance NO/cGMP‐mediated vasodilatation specifically in areas of poor perfusion through the augmentation of V/Q matching, made PDE5 an ideal target for pharmacological intervention.

The first placebo‐controlled study investigating the effect of intravenous sildenafil in PH patients showed significant reductions in pulmonary pressure and pulmonary vascular resistance (Ghofrani, Osterloh, & Grimminger, 2006). Following several case reports and clinical trials confirming the anti‐pulmonary hypertensive effects of sildenafil (Ghofrani, Osterloh, & Grimminger, 2006), this growing body of evidence culminated in the pivotal Sildenafil Use in Pulmonary HypERtension (SUPER‐1) study in 2005: a large, multinational, randomised controlled trial (RCT) which demonstrated improvements in both functional and haemodynamic outcomes from baseline to week 12 following three times daily sildenafil administration (Galiè, Ghofrani et al., 2005). In a long‐term extension study, improvements were maintained after 3 years of therapy (Rubin, Badesch et al., 2011). Oral sildenafil (Revatio®; Pfizer) was approved for the treatment of PAH in both the United States and the European Union in 2005, followed by tadalafil (Adcirca®; Eli Lilly and Company) in 2009.

There have now been several RCTs demonstrating the clear benefits of sildenafil, tadalafil, and vardenafil in PAH, despite a lack of comparative therapeutic data between the agents and the fact that vardenafil is not currently licensed for treatment. A recent Cochrane systematic review and meta‐analysis of 36 studies involving 2,999 patients concluded that patients receiving PDE5Is were more likely to significantly improve their WHO functional class and 6‐min walking distance (6MWD)—a measure of exercise capacity—and were less likely to die, despite an increase in the incidence of side effects such as headache, flushing, and myalgia (Barnes, Brown et al., 2019). Promisingly, these benefits are seen across the PH Group 1 subclass regardless of PAH aetiology, whether idiopathic or associated with connective tissue disease, congenital heart disease, or infection with human immunodeficiency virus (Barnes, Brown et al., 2019). Recently, the AMBITION study demonstrated the rationale for early intervention and targeting multiple pathways in PAH by showing a significant mortality benefit in treatment‐naïve PAH patients receiving long‐term tadalafil–ambrisentan combination therapy compared to monotherapy with either agent (Galiè, Barberà et al., 2015). This evidence of PDE5I–ERA synergy has been adopted in current European guidelines, which favour the use of PDE5I or ERA monotherapy for low‐risk patients (WHO functional class I–III), escalating to the use of combination therapy or intravenous prostacyclin for those at high risk (WHO functional class IV; Hoeper, McLaughlin et al., 2016).

However, existing evidence is insufficient to establish whether pulmonary vasodilatory therapies are safe and effective in other PH subclasses (Hoeper, McLaughlin et al., 2016). Although there is mixed evidence to support PDE5I therapy in PH secondary to left‐heart disease (Group 2; Hutchings, Anderson et al., 2018), there is no clear benefit for use of the agents in PH due to lung disease and/or hypoxia (Group 3) or thrombotic obstruction (Group 4; Barnes, Brown et al., 2019). Indeed, the results seem conflicting even within the same subclass. In PH Group 5 (secondary to systemic or haematological disease), tadalafil significantly improved haemodynamic parameters in β‐thalassaemia intermedia with PH, whereas a trial of sildenafil in PH secondary to sickle cell disease was terminated as a result of a significant increase in clinical worsening and hospitalisation (Barnes, Brown et al., 2019). Similarly, PDE5Is may be harmful in valvular heart disease, a subtype of PH Group 2 (Barnes, Brown et al., 2019). Therefore, whilst PDE5Is have demonstrated significant disease‐modifying properties in PH Group 1 (PAH), the limited and heterogenous nature of trials in other PH subclasses prevents conclusive interpretation of their risk–benefit profile, thus prohibiting their recommendation for the treatment of PH Groups 2 to 5.

Interestingly, therapeutic NO–cGMP intervention with the sGC stimulator riociguat has shown promise in the treatment of chronic thromboembolic PH (CTEPH), the main Group 4 subtype, by reducing pulmonary artery pressure and improving WHO functional class and 6MWD. Although evidence of mortality benefit is lacking, riociguat is currently recommended as a first‐line therapy in the treatment of CTEPH patients who are unfit for pulmonary endarterectomy (Barnes, Brown et al., 2019; Wardle, Seager et al., 2016). Future research into the therapeutic potential of NO–cGMP intervention in PH Groups 2 to 5 should be sufficiently powered and incorporate long‐term follow‐up of clinical, haemodynamic and mortality parameters. This will be aided by further elucidation of the mechanisms of left‐heart disease PH, borderline mean pulmonary arterial pressure increases and mixed clinical phenotypes.

## 6 EMERGING THERAPEUTIC USES OF PDE TYPE 5 INHIBITORS

### 6.1 Cardiovascular disease

CVD now accounts for one third of global mortality—over 90% of these deaths are attributable to three conditions: ischaemic heart disease, stroke and hypertensive heart disease (Joseph, Leong et al., 2017). Research into the cardiovascular benefits of PDE5Is was deferred for several years due to early reports of adverse cardiovascular events in patients prescribed sildenafil (Cheitlin, Hutter et al., 1999). In hindsight, however, this reflected the nature of the patients receiving the drug and long‐term evidence has since established the safety of PDE5Is in both health and CVD (Giuliano, Jackson et al., 2010). Moreover, the demonstration of PDE5 expression in human heart tissue has renewed interest in the use of PDE5Is in this field (Pokreisz, Vandenwijngaert et al., 2009).

#### 6.1.1 Endothelial function

The vascular endothelium, a continuous monolayer of cells lining the entire cardiovascular system, has a key role in a wide range of physiological processes, including regulation of vascular tone and response to inflammation. Endothelial functions are partially mediated by NO–cGMP signalling, deficiency of which is a key feature of endothelial dysfunction (Napoli & Ignarro, 2009), a pro‐thrombotic and pre‐atherosclerotic state which underlies several diseases and is an important adverse modifier of CVD risk and clinical outcomes (Brunner, Cockcroft et al., 2005). As highlighted in a recent review (Schwartz, Jackson et al., 2013), both single‐use and long‐term administration of PDE5Is in patients with high cardiovascular risk have been shown to ameliorate endothelial function parameters, reduce plasma concentrations of ET‐1 and endothelial inflammatory mediators and increase levels of circulating endothelial progenitor cells. Promisingly, several studies have indicated that PDE5I‐mediated improvements in endothelial function may be sustained for weeks to months after discontinuation of long‐term treatment (McLaughlin, Lytvyn et al., 2014), signifying that, in addition to their acute haemodynamic effects, the agents may produce fundamental modifications at a cellular level, which may explain the therapeutic effects of PDE5Is in CVD, CKD and diabetes mellitus, as discussed below. Moreover, limited clinical data suggest that sildenafil provides sustained pharmacological preconditioning of human endothelium which may protect against the adverse effects of ischaemia–reperfusion on vascular function (McLaughlin, Lytvyn et al., 2014).

#### 6.1.2 BP homeostasis

Hypertension is the most important global risk factor for CVD (Olsen, Angell et al., 2016). NO–cGMP signalling is central to the regulation of BP homeostasis in hypertension through its effects on vascular tone, the sympathetic nervous system, and renal salt and water handling (Mergia & Stegbauer, 2016). The vasodilatory effects of PDE5Is provide a rationale for use in systemic arterial hypertension: Acutely, single doses of PDE5Is result in transient reductions of both peripheral and aortic BP, although some variation in effect exists between health and hypertension and between different PDE5Is (Jackson, Benjamin et al., 1999; Kloner, Mitchell, & Emmick, 2003; Mahmud, Hennessy, & Feely, 2001; Pomara, Morelli et al., 2004). In a landmark study, regular sildenafil monotherapy for 16 days significantly reduced ambulatory BP by around 6 mmHg in patients with untreated hypertension compared to placebo (Oliver, Melville, & Webb, 2006). These results were further supported by the demonstration of significant ambulatory BP reductions in hypertensive patients of around 6 mmHg following 28 days of monotherapy with PF‐00489791, a highly specific and long‐acting PDE5I, compared to placebo (Wolk, Smith et al., 2009). In both studies, PDE5I use was safe and well‐tolerated.

Alternative uses for PDE5Is are also being explored in the field of treatment‐resistant hypertension (RHTN). Several studies have demonstrated the safety of PDE5I use in combination with other antihypertensive therapies (Kloner, Brown et al., 2001; Kloner, Mitchell et al., 2003; Nehra, 2009; Pickering, Shepherd et al., 2004). Concomitant administration of organic nitrates and PDE5Is is currently contraindicated due to the acute risk of hypotension (Webb, Freestone et al., 1999), although more recent studies have suggested that the interaction between the two agents is confined to within 6 hr post‐sildenafil administration (Oliver, Kerr, & Webb, 2009; Parker, Bart et al., 2007). Nevertheless, the synergistic hypotensive interaction occurring with concomitant PDE5I and nitrate administration may yet be exploited therapeutically, as demonstrated in a proof of concept study in which sildenafil and isosorbide mononitrate combination therapy in RHTN patients produced an additional BP reduction superior to the two agents individually, suggesting a novel approach for the treatment of RHTN (Oliver, Dear, & Webb, 2010). Recently, a crossover trial showed that acute administration of sildenafil reduced mean BP and improved diastolic dysfunction parameters in patients with RHTN by facilitating a reduction in total peripheral resistance (Quinaglia, de Faria et al., 2013). Notably, exploratory analysis revealed that NOS3 polymorphism between subjects may have played a role in modulating the sildenafil response. PDE5 activation has also been implicated in angiotensin II‐dependent hypertension (Ramseyer, Ortiz et al., 2016) and in experimental models, PDE5 inhibition has been demonstrated to reduce angiotensin II levels and restore the baroreflex in renovascular hypertension (Cavalcanti, Alves et al., 2016; Dias, Rodrigues et al., 2014).

#### 6.1.3 Cardioprotection

The NO–cGMP signalling pathway has been identified as a survival signal in ischaemic heart disease (Burley, Ferdinandy, & Baxter, 2007), facilitating its essential cardioprotective effects via PKG‐dependent phosphorylation of ERK and GSK3B, increased generation of NO by NOS2/3, activation of PKC and opening of mitochondrial ATP‐sensitive potassium channels (mitoK_ATP_), which stabilise the membrane potential to allow ATP synthesis and calcium ion transport (Inserte & Garcia‐Dorado, 2015). Preclinical studies have successfully shown that PDE5Is can enhance this pathway in ischaemia–reperfusion injury and myocardial infarction (MI) by increasing levels of inducible and NOS3 activity in the heart (Das, Xi, & Kukreja, 2005), augmenting mitoK_ATP_ opening via PKG‐dependent mechanisms (Ockaili, Salloum et al., 2002) and delaying normalisation of intracellular acidosis (Inserte, Barba et al., 2011). These effects are independent of vasoactive properties, mediated instead through direct myocardial anti‐inflammatory and anti‐apoptotic processes (Das, Xi, & Kukreja, 2005; Salloum, Abbate et al., 2008). These mechanisms of post‐ischaemic protection have been linked to significant reductions in infarct size and cardiomyocyte death in animals, regardless of the timing of PDE5I administration, whether given minutes to weeks in advance of the ischaemic insult (preconditioning; Jamnicki‐Abegg, Weihrauch et al., 2007; Ockaili, Salloum et al., 2002; Salloum, Abbate et al., 2008; Wang, Fisher et al., 2008), or indeed immediately prior to or during coronary reperfusion (postconditioning; Ebner, Ebner et al., 2013; Madhani, Hall et al., 2010; Salloum, Takenoshita et al., 2007). However, there is no experimental evidence on the administration of PDE5I during reperfusion following more than half an hour of ischaemia, which may more accurately reflect clinical practice, associated with delayed patient presentation and longer inter‐hospital transfer times.

Similarly, the rationale for PDE5I‐mediated augmentation of the cGMP–PKG pathway in select cases of MI and heart failure has been fully appraised in a recent review (Hutchings, Anderson et al., 2018). The authors conclude that PDE5Is may ameliorate the clinical condition of patients with heart failure with reduced ejection fraction (HFrEF) through improvements in the pulmonary circulation, cardiac remodelling and diastolic function. In heart failure with preserved ejection fraction (HFpEF), however, these beneficial observations are largely offset by a reduction in cardiac contractility. Therefore, PDE5I use in heart failure may be limited to HFrEF and HPpEF with associated PH or right ventricular systolic impairment, although the underlying signalling pathways for this variation in effect are not well understood. In future studies, careful patient stratification by phenotype is crucial. The cardioprotective effects of PDE5Is are also being investigated in chemotherapy‐induced cardiotoxicity, where adjunctive treatment has been shown to reduce apoptosis and improve left ventricular function without interfering with the chemotherapeutic benefits of doxorubicin (Fisher, Salloum et al., 2005; Koka, Das et al., 2010).

#### 6.1.4 CNS effects

The NO–cGMP signalling pathway is involved also in several physiological and pathological cerebral functions. PDE5 is widely expressed in the CNS in neural and vascular tissue (Zhang, Zhang, & Chopp, 2013), and PDE5Is have been shown to cross the blood–brain barrier to directly affect brain tissue (García‐Barroso, Ricobaraza et al., 2013; Gómez‐Vallejo, Ugarte et al., 2016), perhaps by enhancing angiogenesis and neurogenesis (Zhang, Zhang, & Chopp, 2013), highlighting the potential for therapeutic intervention in both ischaemic stroke and neurodegenerative disorders. However, the few studies investigating these effects in humans are limited by their focus on cerebral blood flow (Pauls, Moynihan et al., 2018), which is not a key surrogate for brain function. Moreover, their findings are inconclusive, and there is no attempt to correlate changes in blood flow to improvements in cognitive function. Therefore, the potential relevance of PDE5I therapy to cerebrovascular disease remains uncertain. Nevertheless, recent developments in our understanding of the neurophysiological roles of NO–cGMP signalling, reviewed elsewhere (Garthwaite, 2019), may yield novel interventional opportunities in stroke and other neurological disorders.

#### 6.1.5 Peripheral circulation

Reports of improvements in walking distance in patients with both limb and buttock claudication following sildenafil therapy support the potential for PDE5I therapy to benefit patients with peripheral arterial disease (Omarjee, Le Pabic et al., 2019). In a similar fashion to the angiogenic potential of PDE5Is demonstrated in experimental stroke models, preclinical studies have demonstrated improvements in vascular perfusion, tissue blood flow, and vascular density with sildenafil (Senthilkumar, Smith et al., 2007) and vardenafil (Sahara, Sata et al., 2010) in a mouse model of unilateral hindlimb ischaemia. Recently, a prospective crossover study of 12 patients reported a significant improvement in maximal walking time with a single oral dose of sildenafil, although this did not impact pain‐free walking time or skin tissue oxygenation during exercise (Omarjee, Le Pabic et al., 2019). Further prospective studies investigating the impact of PDE5I treatment on clinical outcomes are ongoing (Table 3).

The vasodilatory and endothelial effects of PDE5Is have also shown promise in the treatment of Raynaud's phenomenon (RP). RP is a condition characterised by episodic cold‐ or stress‐induced ischaemic vasospastic episodes affecting the digital arteries and arterioles leading to ulcerations and necrosis and is either idiopathic (primary RP) or due to connective tissue disease (secondary RP). A recent meta‐analysis of six RCTs that included 244 patients with secondary RP, predominantly due to scleroderma, found that PDE5I therapy moderately reduced the daily frequency and duration of vasospastic attacks and promoted visible healing of ulcers (Roustit, Blaise et al., 2013) and a prospective crossover study suggests that these beneficial effects also extend to primary RP (Caglayan, Huntgeburth et al., 2006). These findings suggest that PDE5I therapy may have an equivalent effect on vasospastic attack frequency to first‐line treatment with calcium channel blockers (CCBs) and is more successful at reducing attack frequency and promoting ulcer healing than ERAs, an effective and approved treatment for reducing digital ulceration in scleroderma but which has inconsistent or minimal effects on other clinical parameters and is of uncertain benefit in primary RP (Roustit, Blaise et al., 2013). Moreover, recent interventional trials (Table 3) have demonstrated a significant improvement in digital blood flow in patients with secondary RP following the use of both oral and topical sildenafil preparations (Andrigueti, Ebbing et al., 2017; Wortsman, Del Barrio‐Díaz et al., 2018), and a recent RCT showed that chronic treatment with sildenafil improved healing of digital ulcers secondary to systemic sclerosis (Hachulla, Hatron et al., 2016).

Although there has been little research into the therapeutic efficacy of PDE5Is in primary RP, subgroup analysis of a recent RCT investigating chronic vardenafil use in 53 adults with mixed RP revealed an increased efficacy in patients with primary RP (Caglayan, Axmann et al., 2012). However, significant clinical improvement was not replicated in a series of *n*‐of‐1 trials of increasing sildenafil dosage in 38 patients with mixed RP (68% of whom had primary RP), which demonstrated a markedly heterogenous response (Roustit, Giai et al., 2018). Therefore, although PDE5I therapy may represent a promising intervention for improving the microcirculation and clinical outcomes of patients with RP, further research with clearer stratification of RP causality, direct comparison with oral CCBs, and standardised tools for assessment of digital blood flow is necessary to clarify the efficacy and safety of these agents and determine which group of patients may benefit most from treatment.

### 6.2 Chronic kidney disease

CKD is a global public health problem characterised by a reduced GFR and/or evidence of kidney damage (Levin, Stevens et al., 2013). High BP and diabetes mellitus are major contributors to the development of CKD and both are associated with an increased risk of CVD. In addition, CKD is a major independent risk factor for incident CVD, which remains the main cause of death in these patients (Go, Chertow et al., 2004). The incidence and prevalence of CKD is rising, and its progressive clinical course is characterised by high rates of morbidity and mortality (Hoerger, Simpson et al., 2015).

The past decade has seen substantial progress in understanding the pathogenesis of CKD, with compelling evidence to suggest that the NO–cGMP pathway plays an important role in the maintenance of renal perfusion and glomerular filtration and that CKD is a state of systemic NO deficiency (Brown, Dhaun et al., 2014). Recently, the administration of sildenafil in a 5/6 renal ablation model has been shown to suppress vascular smooth muscle cell proliferation and inflammation and, subsequently, prevent progression of histological and functional damage in the remaining glomeruli (Tapia, Sanchez‐Lozada et al., 2012). Moreover, preclinical studies have indicated the potential for PDE5Is to reverse podocyte dysfunction and normalise proteinuria, independently of BP (Fang, Radovits et al., 2012; Hall, Rowell et al., 2014).

Although the first clinical trial to evaluate the potential therapeutic benefits of PDE5Is in CKD demonstrated that chronic sildenafil monotherapy significantly reduced microalbuminuria in patients with diabetic nephropathy (DN; Grover‐Páez, Rivera, & Ortíz, 2007), there were a number of methodological limitations which prohibited conclusive interpretation of the results (Brown, Dhaun et al., 2014). In order to address these issues, Pfizer launched an RCT investigating the use of PF‐00489791 for the treatment of overt DN (Scheele, Diamond et al., 2016). The inclusion criteria dictated a history of at least 3 months of persistent, overt macroalbuminuria—defined as a urinary albumin/creatinine ratio (UACR) of over 300 mg·g^−1^ — despite treatment with ACE inhibitor (ACEI) or angiotensin receptor blocker (ARB) in combination with antihypertensive and antidiabetic medications. Eligible participants had an estimated GFR (eGFR) of 25–59 ml·min^−1^ per 1.73 m^2^ (CKD stage G3 or G4) and were randomly assigned to receive either oral PF‐00489791 therapy or placebo once daily for 12 weeks. Remarkably, in the context of treatment‐resistant macroalbuminuria, the PDE5I group demonstrated a significant reduction in albuminuria as early as the third week of therapy. At the end of the 12‐week treatment period, patients receiving PF‐00489791 had a significant, BP‐independent, 16% reduction in UACR compared with placebo. However, this reduction in UACR was reversible and was non‐significant at 16 weeks, 4 weeks after the end of treatment. Although there was no difference in eGFR between treatment and placebo groups throughout the treatment period, this would be unlikely within the short timeframe. Interestingly, there was no initial short‐term fall in eGFR with PDE5I as is usually observed with ACEI/ARB treatment.

These encouraging results suggest that enhancing NO–cGMP signalling confers renoprotective effects and improvements in kidney function, possibly as a result of both haemodynamic and intrarenal anti‐inflammatory, anti‐proliferative and antioxidant mechanisms (Brown et al., 2014). However, no ongoing interventional studies on the long‐term benefits of PDE5I therapy in renal diseases could be identified (Table 3). We eagerly await the results of two recently completed studies evaluating the use of PDE5Is in renal disease to determine whether further research is merited (NCT02414204 and NCT01960153).

### 6.3 Diabetes mellitus

Type 2 diabetes mellitus (T2DM) is characterised by hyperglycaemia and results from a combination of peripheral insulin resistance and progressive pancreatic β‐cell dysfunction. These pro‐inflammatory states confer higher rates of incident hypertension and cardiovascular risk. Indeed, CVD accounts for two‐thirds of deaths in diabetic patients, with renal sequelae compounding the high rates of morbidity and mortality of this condition (Wang, Hess et al., 2016). It is well recognised that achieving tight glycaemic control must be combined with appropriate prevention and management of CVD and renal disease in order to maximise positive outcomes. Recent data suggest the potential for PDE5Is to modulate the macrovascular, microvascular and metabolic complications associated with diabetes mellitus.

#### 6.3.1 Macrovascular effects

In a landmark study, a population of 5,956 men diagnosed with T2DM were monitored over a median period of 7 years to determine the effect of PDE5I use on their overall survival (Anderson, Hutchings et al., 2016). Compared to a random sample of non‐users from the same background population, men prescribed a PDE5I had a significant 31% lower risk of all‐cause mortality. This reduction in mortality remained statistically significant after adjusting for known risk modifiers including age, eGFR, smoking status, prior history of CVD or hypertension, known MI, systolic BP, lipid profile, number of prescriptions and Townsend deprivation index, as well as use of cardioprotective agents such as statins, metformin, aspirin and ß‐adrenoceptor antagonists (beta‐blockers), although life course socio‐economic position, compliance and doctor selection practices could not be adjusted for. Significant reductions were also observed in age‐adjusted incidence and mortality rates of acute MI in patients with a history of PDE5I use when compared to subjects never prescribed this class of drug. Notably, ED—the most common indication for PDE5I use—is a strong predictor of subsequent ischaemic heart disease (Gazzaruso, Solerte et al., 2008), suggesting that the potential protective effects of PDE5Is on cardiac tissue and vasculature may offset the pre‐existing additional CVD risk associated with this condition. Moreover, in an RCT of 59 T2DM men with diabetic cardiomyopathy, 3 months of sildenafil treatment led to significant improvements in cardiac indices compared to placebo (Giannetta, Isidori et al., 2012). Notably, these men were also on optimal anti‐hyperglycaemic, ACEI/ARB, ß‐adrenoceptor antagonists and CCB therapies, highlighting the additional therapeutic potential of PDE5Is in the management of diabetic complications.

Although these are promising findings, the exact mechanism by which PDE5Is may improve survival in diabetic patients is unclear but may pertain to the ability of PDE5Is to ameliorate, or prevent the development of, cardiovascular and renal pathology in both diabetic and non‐diabetic subjects.

#### 6.3.2 Microvascular effects

The cardioprotective effects of PDE5Is may also extend to the diabetic myocardium. T2DM is associated with higher levels of inflammation and oxidative stress in the heart, leading to myocardial injury and worse outcomes compared to healthy heart tissue (Ray, Cannon et al., 2007). Also, the diabetic myocardium is more susceptible to ischaemia–reperfusion injury and is resistant to several cardioprotective modalities (Miki, Itoh et al., 2012). With this background, it is promising that, in T2DM mice exposed to ischaemia–reperfusion events, chronic tadalafil treatment significantly reduced infarct size, improved glucose metabolism, and ameliorated inflammatory and oxidative profiles in the diabetic myocardium (Koka, Das et al., 2013; Koka, Xi, & Kukreja, 2012; Varma, Das et al., 2012). Moreover, an RCT of 54 male patients with T2DM recently demonstrated significant, sustained improvements in endothelial parameters following chronic vardenafil use (Santi, Granata et al., 2016). Interestingly, this was also accompanied by restoration of testosterone levels in men with hypogonadism as early as 1 week into the treatment protocol, unrelated to erectile function scoring and, therefore, possibly independent of sexual activity.

#### 6.3.3 Metabolic effects

Clinical trials have suggested that PDE5Is may influence glucose metabolism. In addition to the reductions in albuminuria seen in studies evaluating the renoprotective effects of these agents, daily PDE5I therapy for 1–4 months produced significant decreases in mean glycated Hb (HbA1c) of 0.3–0.59% from baseline (Grover‐Páez, Rivera et al., 2007; Scheele, Diamond et al., 2016), representing a reduction of hyperglycaemia comparable to that observed with established oral antidiabetic agents administered for longer treatment periods (0.5–1.25%; Sherifali, Nerenberg et al., 2010). Given the linear association between HbA1c and macrovascular disease, with an estimated 11–16% increase in cardiovascular events for every 11 mmol·mol^−1^ (1%) rise in HbA1c (Wang, Hess et al., 2016), these findings may have important implications for the management of insulin resistance in diabetes and obesity.

Insulin has recognised vasodilatory properties mediated by the up‐regulation of NOS3 expression and activity (Kuboki, Jiang et al., 2000). Insulin resistance has been shown to inhibit this pathway by reducing endothelial NO bioavailability (Wang, Hess et al., 2016). This disruption of NO–cGMP signalling and the resulting endothelial dysfunction has been shown to precede the development of inflammation and insulin resistance across muscle, liver and adipose tissue in mice with diet‐induced obesity (Kim, Pham et al., 2008; Rizzo, Maloney et al., 2010; Tateya, Rizzo et al., 2011). PDE5Is have proven anti‐inflammatory effects in diabetes‐induced endothelial dysfunction (Schwartz, Jackson et al., 2013) and recent evidence suggests that PDE5Is have additional direct effects on glucose metabolism: Significant improvements in insulin sensitivity and glucose uptake have been demonstrated in the muscles of mice fed a high‐fat diet following 12‐week sildenafil treatment, suggesting that PDE5I therapy may improve energy balance and prevent insulin resistance (Ayala, Bracy et al., 2007). Similar improvements in glucose uptake with PDE5I use have also been reported in experimental animal (Sabatini, Sgrò et al., 2011; Zhang et al., 2010) and human (Jansson, Murdolo et al., 2010) studies, further suggesting a link between NO–cGMP signalling and insulin action. Although insulin treatment does not induce NO production in insulin resistance (Salt, Morrow et al., 2003), chronic sildenafil treatment has been shown to enhance NOS3 activity in insulin resistance conditions (Mammi, Pastore et al., 2011), suggesting potential for sildenafil to improve vascular function in diabetes mellitus. Finally, others have demonstrated that sildenafil may promote adipogenesis via PKG‐mediated up‐regulation of lipid accumulation and insulin sensitivity in adipocytes (Zhang, Ji et al., 2010). Indeed, two RCTs have recently reported an improvement in oral disposition index (a composite measure of insulin sensitivity and secretion) in patients with the metabolic syndrome following long‐term PDE5I therapy, reflecting an overall improvement in β‐cell function (Ramirez, Nian et al., 2015; Santi, Granata et al., 2016).

Therefore, PDE5I treatment may affect outcomes in patients with diabetes mellitus through reversal of both vascular dysfunction, which is associated with increased CVD risk, and metabolic impairment. Collectively, the available data provide a strong rationale for further research into the therapeutic targeting of the NO–cGMP pathway for the management of T2DM and obesity, although the promise shown in recent studies with ERAs (Dhaun & Webb, 2019) and sodium–glucose cotransporter 2 (SGLT2) inhibitors (Perkovic, Jardine et al., 2019) in patients with T2DM and renal disease serves to make this a competitive field, to the benefit of future patients.

## 7 PHARMACOLOGY AND SAFETY PROFILE OF PDE TYPE 5 INHIBITORS

Although all PDE5Is employ the same mechanism of action and exhibit similar therapeutic efficacy and safety (Jannini, Isidori et al., 2009), they are distinguished on the basis of their pharmacokinetic and pharmacodynamic properties (Hatzimouratidis & Hatzichristou, 2005).

### 7.1 Pharmacokinetics

The primary pharmacokinetic variables which determine PDE5I administration are bioavailability, maximum plasma concentration after an oral dose (*C*_max_), time to achieve maximum plasma concentration (*T*_max_) and terminal half‐life (*t*_½_; Table 1). All marketed PDE5Is have a high volume of distribution into the vascular space, tissues and binding by extravascular proteins such as albumin (Mehrotra, Gupta et al., 2007). Although comparative bioavailability data between the agents are lacking, this can be estimated by quantifying the area under the plasma drug concentration‐time curve (AUC). Notably, vardenafil has a significantly lower AUC than sildenafil and tadalafil, which may explain its comparatively lower *C*_max_. However, it is not possible to correlate these kinetics with the clinical dosing regimens of the other agents from the information that is publicly available.

**TABLE 1:** PDE5I pharmacokinetic and pharmacodynamic comparison

PDE5I	Pharmacokinetics					Pharmacodynamics			
	Dose (mg)	*T*_max_ (hr)	*t*_½_ (hr)	*C*_max_ (ng·ml^−1^)	AUC (ng × hr·ml^−1^)	MW (g·mol^−1^)	MF	IC_50_ for PDE5 (nmol·L^−1^)	PDE selectivity
Sildenafil	100	0.95	3.98	514	1,670	475	C_22_H_30_N_6_O_4_S	3.7	Low activity against PDE6; very low activity against PDE1.
Tadalafil	20	2	17.5	378	8,066	389	C_22_H_19_N_3_O_4_	1.8	Low activity against PDE11; very low activity against PDE6.
Vardenafil FCT	20	0.66	3.9	20.9	74.5	488	C_23_H_32_N_6_O_4_S	0.091	Low activity against PDE6; very low activity against PDE1.
Avanafil	200	0.75	5.1	2,920	8,490	484	C_23_H_26_CIN_7_O_3_	5.2	Highly selective for PDE5.
Udenafil	200	0.76	9.88	1,137	7,898	517	C_25_H_36_N_6_O_4_S	8.25	Comparable to sildenafil for PDE5.
Mirodenafil	100	1.4	2.5	2,989	7,907	532	C_26_H_37_N_5_O_5_S	0.33	Comparable to sildenafil for PDE5.
Lodenafil	160	1.2	2.4	157	530	1,035	C_47_H_62_N_12_O_11_S_2_	0.015	Low activity against PDE1 and PDE6.
PF‐00489791	20	1.1–3.5	11.9–15.7	1,570–1,630	N/A	477	C_20_H_28_N_8_O_4_S	0.71	Highly selective for PDE5.

*Note.* Data sourced from Clerin, Gale, & Tamimi (2014) and Hatzimouratidis, Salonia et al. (2016).

Abbreviations: AUC, area under the plasma drug concentration‐time curve; *C*_max_, maximum plasma serum concentration; FCT, film‐coated tablet; IC_50_, half maximal inhibitory concentration; MF, molecular formula; PDE5I, PDE type 5 inhibitor; *t*_½_, terminal plasma half‐life; *T*_max_, time taken to reach the maximum plasma concentration.

Sildenafil, udenafil, vardenafil and avanafil have similar *T*_max_ values and thus comparable onset of action after oral administration (30–60 min), with the latter two having the fastest onset: Vardenafil, in both formulations, acts within 30 min of intake whereas avanafil is the only PDE5I approved for use 15–30 min before sexual intercourse in the treatment of ED. In comparison, mirodenafil and lodenafil have slightly higher *T*_max_ values, with limited published data suggesting that these have a similar onset of action to sildenafil, whereas tadalafil has the highest *T*_max_, reaching peak plasma concentration after 30–120 min (Hatzimouratidis, Salonia et al., 2016).

Tadalafil and udenafil have the highest *t*_½_ of marketed PDE5Is, although it is unclear whether this is due to either delayed absorption or degradation. Clinically, this translates to a longer duration of therapeutic effect (up to 36 hr for tadalafil and 12 hr for udenafil). Tadalafil is uniquely unaffected by dietary intake, enabling once daily dosing such as that required for the management of lower urinary tract symptoms in benign prostatic hyperplasia—an indication for which tadalafil was licensed in 2011 and which is further explored in a recent review (Andersson, 2018). Avanafil has the shortest duration of action (up to 6 hr), with sildenafil, vardenafil, mirodenafil and lodenafil lasting slightly longer (up to 10–12 hr; Hatzimouratidis, Salonia et al., 2016).

PDE5Is are rapidly metabolised by the cytochrome P450 enzyme family (CYP450) in the liver, chiefly through the CYP3A system with contributions from CYP2C9, CYP2C19, and CYP2D6 pathways (Mehrotra, Gupta et al., 2007), and systemic clearance of sildenafil and vardenafil, but not tadalafil, is reduced by mild and moderate hepatic impairment (Muirhead, Wilner et al., 2002). Both sildenafil and vardenafil have active metabolites which significantly contribute to their pharmacological activity (Mehrotra, Gupta et al., 2007). Although PDE5Is are largely faecally excreted, undergoing minimal renal clearance due to their extensive tubular reabsorption, the secondary effects of renal impairment may affect the clearance of sildenafil through alterations in protein binding or inflammatory protein synthesis (Muirhead, Wilner et al., 2002). Therefore, patients with severe hepatic or renal impairment should be prescribed a lower starting dose of PDE5Is and titrated up as appropriate.

### 7.2 Pharmacodynamics

The available PDE5Is differ in their affinity and selectivity for the PDE5 isoform, with consequent effects on their tolerability and safety profile. The biochemical affinity of the various agents is quantified by the half maximal inhibitory concentration (IC_50_), which represents the concentration of the agent that is required to inhibit 50% of PDE5 activity in vitro. Affinity is an inverse measure of potency, such that an agent with a higher IC_50_ requires a lower dose to reach therapeutic effect. Selectivity refers to the ability of a PDE5I to preferentially bind PDE5 over other PDEs (Table 1). The cross‐reactivity of PDE5Is has important implications for the tolerability of the agent as the ubiquitous presence of PDEs in the body means that non‐selective inhibition may result in wide‐ranging systemic effects.

All agents are generally well‐tolerated (Table 2) and share similar transient, mild side effects, such as facial flushing, headache, dyspepsia, nasal congestion and dizziness. Tadalafil has also been associated with myalgia and back pain in up to 6% of patients. However, sildenafil and, to a lesser extent, vardenafil have demonstrated cross‐reactivity with PDE1 and PDE6. PDE6 is highly expressed in retinal photoreceptors, where its inhibition causes transient blue/green colour vision disturbances which coincide with peak plasma sildenafil concentrations (Hatzimouratidis & Hatzichristou, 2005). PDE5Is also show some cross‐reactivity with PDE1. PDE1 is highly expressed in vascular smooth muscle, where its inhibition results in vasodilatation and BP reduction, as demonstrated recently in rats with novel PDE1‐inhibitors (Laursen, Beck et al., 2017). It has been suggested that the higher incidence of facial flushing following sildenafil and vardenafil administration, compared with tadalafil and avanafil, may be the result of greater PDE1 cross‐reactivity (Kouvelas, Goulas et al., 2009). Finally, tadalafil has been shown to inhibit the activity of PDE11, an isozyme which is expressed in the mammalian testes and prostate, although no impact on reproductive function has been reported with PDE5I use (Hatzimouratidis & Hatzichristou, 2005). Indeed, a large meta‐analysis suggests that chronic PDE5I use may have a beneficial effect on spermatogenesis, although the improvements with tadalafil are less than would be expected given its long duration of action (Tan, Liu et al., 2017). Although quantitative isozyme affinity and selectivity data for udenafil, mirodenafil and lodenafil are not publicly available, these are said to have a comparable selectivity profile to sildenafil, which may explain their similar side effect profile. In comparison, avanafil and PF‐00489791 are newer agents with improved PDE5 selectivity and seemingly favourable tolerability (Table 2), particularly with respect to visual disturbances (Wang, Burnett et al., 2012).

**TABLE 2:** Reported PDE5I side effect incidence

PDE5I	Observed incidence of side effect (%)									
	Headache	Facial flushing	Dyspepsia	Rhino‐sinusitis	Back pain	Myalgia	Abnormal vision	Red eye	Chest discomfort	Peripheral oedema
Sildenafil	19	14	9	5	0	0	6	0	0	0
Tadalafil	21	5	17	5	9	7	0	0	0	0
Vardenafil	16	12	4	10	0	0	<2	0	0	0
Avanafil	7–8	3–5	2	2	<2	0	0	0	0	0
Udenafil	2–9	11–23	0	4–7	0	0	0	4–7	0–5	0
Mirodenafil	8–11	10–16	3	0	0	0	0	3–4	0–3	0
Lodenafil	15–22	5–6	5–22	5–11	0	0	5–6	0	0	0
PF‐00489791	5–21	0	3–15	0	0–12	0	0	0	0	1–9

*Note.* Data sourced from Glina, Toscano et al. (2009); Hatzimouratidis and Hatzichristou (2005); Limin, Johnsen, & Hellstrom (2010); Paick, Ahn et al. (2008); Paick, Kim et al. (2008); Scheele et al. (2016); Wang, Burnett et al. (2012); and Wolk, Smith et al. (2009).

Abbreviation: PDE5I, PDE type 5 inhibitor.

In theory, agents with improved PDE5 selectivity may achieve comparable efficacy with minimal systemic consequences by limiting PDE1/PDE6 cross‐reactivity. However, the highly selective agents PF‐00489791 and PF‐03049423 have recently been withdrawn from several clinical trials investigating their use in various cardiovascular indications (https://adisinsight.springer.com/drugs/800025495), despite a lack of safety concerns and the former's previously discussed efficacy in hypertension and CKD. Although PF‐03049423 was not found to be effective in improving outcomes following ischaemic stroke (Di Cesare, Mancuso et al., 2016), the rationale behind the withdrawal of PF‐00489791 is not publicly available. Similarly, research into KD027/SLx‐2101, a PDE5I which was uniquely converted into a long‐acting active metabolite and had been in the final stages of development for the treatment of hypertension (NCT00562549 and NCT00562614; Hatzimouratidis & Hatzichristou, 2008), has also been suspended following the closure of its sponsor company (Professor I. Wilkinson, personal communication, September 16, 2019). In combination with the recent surge in registered trials of sildenafil and tadalafil in several cardiovascular conditions (Table 3), these developments indicate a market shift towards re‐purposing already established PDE5Is. Although the reasons for this are unclear, it is possible that these novel PDE5Is may not have achieved adequate therapeutic efficacy for their development to be financially sustainable given recent advancements in the field of cardiovascular therapeutics, particularly with regard to the extensive and evolving therapeutic profile of SGLT2‐inhibitors.

**TABLE 3:** Ongoing interventional clinical trials of PDE5Is beyond ED and PH

Drug(s) tested	Sponsor	Target group	Stage of development (status; clinical identifier)	Results of clinical trial/reason for termination
*Hypertension*				
Sildenafil	University of Edinburgh, UK; Pfizer, USA	Primary hypertension	Phase IV (completed 2005; NCT00479908)	Significant ambulatory BP reduction compared to placebo (Oliver, Melville, & Webb, 2006).
PF‐00489791	Pfizer, USA	Primary hypertension	Phase II (completed 2008; NCT00422461)	Significant ambulatory BP reduction compared to placebo (Wolk, Smith et al., 2009).
Sildenafil	University of Campinas, Brazil	RHTN	Not applicable (completed 2012; NCT01392638)	Significant improvement in diastolic function and BP compared to placebo (Quinaglia, de Faria et al., 2013).
Tadalafil	University of Campinas, Brazil	Left ventricular function in RHTN	Not applicable (completed 2012; NCT01743911)	Significant improvement in diastolic function compared to placebo. No significant change in BP (Santos, de Faria et al., 2014).
*Cardiac*				
Tadalafil	New England Research Institutes; NHLBI; Massachusetts General Hospital, USA	HFrEF	Phase III (terminated 2015; NCT01910389)	Terminated by funding agency.
Udenafil	Seoul National University Hospital; Dong‐A Pharm, South Korea	HFrEF	Phase III (terminated 2014; NCT01646515)	Substantial benefit was observed in the active treatment group.
Sildenafil	Massachusetts General Hospital, USA	HFrEF	Phase III (completed 2006; NCT00309790)	Improved cardiac indices (Malhotra, Dhakal et al., 2016).
Sildenafil	University of Milan, Italy	HFpEF	Phase II/III (completed 2009; NCT00781508 and NCT01156636)	Improved cardiac indices and clinical status (Guazzi, Vicenzi et al., 2011).
Sildenafil	University of Milan, Italy	HFrEF	Phase II (completed 2010; NCT01185925)	Not reported.
Vardenafil	McGuire Research Institute, USA	IRI after cardiac surgery	Phase I (completed 2011; NCT01260285)	Not reported.
Sildenafil	Duke University; NHLBI; Pfizer, USA	HFpEF	Phase III (completed 2012; NCT00763867)	No effect on exercise capacity or clinical status (Redfield, Chen et al., 2013).
Sildenafil	Rigshospitalet, Denmark	HFpEF after MI	Phase IV (completed 2012; NCT01046838)	No improvement in filling pressure. Significant improvements in cardiac indices, exercise capacity and diastolic BP (Andersen, Ersbøll et al., 2013).
Udenafil	Seoul National University Hospital; Dong‐A Pharm, South Korea	HFpEF	Phase III (completed 2013; NCT01599117)	Not reported.
Sildenafil	Hospital Ana Nery, Brazil	Unspecified HF	Phase II/III (completed 2013; NCT01936350)	No effect on cardiac indices (Fernandes, Andrade et al., 2015).
Tadalafil ± Nesiritide	Mayo Clinic; NCRR; NHLBI, USA	Preclinical HF, expected renal function improvement	Phase I/II (completed 2014; NCT01544998)	Not reported.
Sildenafil	University Medical Center Groningen, Netherlands; Pfizer, USA	HFpEF	Phase III (completed 2014; NCT01726049)	No effect on cardiac indices or clinical status (Liu, Hummel et al., 2017).
Sildenafil	Pfizer, USA	HFrEF	Phase III (ongoing; NCT01616381 and NCT03460470)	Ongoing.
Udenafil	Mezzion Pharma Co. Ltd, South Korea; NHLBI, USA	Congenital heart defects	Phase III (ongoing; NCT02741115)	Ongoing.
Sildenafil	University of Calgary, Canada; Pfizer, USA	RHF	Phase III (ongoing; NCT03356353)	Ongoing.
*Stroke*				
PF‐03049423	Pfizer, USA	Acute ischaemic stroke	Phase II (terminated 2016; NCT01208233)	Interim analysis demonstrated futility. No reported safety concerns (Di Cesare, Mancuso et al., 2016).
Sildenafil	University of Utah, USA	Subacute ischaemic stroke	Phase I (terminated 2017; NCT02628847)	Recruitment failure.
Sildenafil	Henry Ford Hospital, USA	Subacute ischaemic stroke	Phase I (terminated 2011; NCT00452582)	Recruitment failure.
Tadalafil	Herlev Hospital, Denmark	Cerebral small vessel disease	Phase II (completed 2017; NCT02801032)	Not reported.
Sildenafil	University of Oxford, UK	Cerebral small vessel disease	Phase II (ongoing; NCT03855332)	Ongoing.
*Peripheral circulation*				
Vardenafil	University of Cologne, Germany	Mixed RP	Phase II/III (completed 2010; NCT01291199)	Significant improvement in digital blood flow and clinical symptoms compared to placebo (Caglayan, Axmann et al., 2012).
Tadalafil	Sanjay Gandhi Postgraduate Institute of Medical Sciences, India	Treatment‐resistant secondary RP	Phase III (completed 2010; NCT00626665 and NCT01117298)	Significant improvement in clinical symptoms, quality of life, digital ulcer frequency, and healing with add‐on PDE5I therapy compared to placebo (Shenoy, Kumar et al., 2010).
PF‐00489791	Pfizer, USA	Mixed RP	Phase II (completed 2011; NCT01090492)	Not reported. Trials discontinued.
Udenafil	Seoul National University Hospital, South Korea	Secondary RP	Phase II/III (completed 2011; NCT01280266)	Significant improvement in digital blood flow compared to amlodipine. Similar efficacy in reducing RP attacks (Lee, Park et al., 2013).
Sildenafil	Lille University Hospital, France	Secondary RP	Phase III (completed 2013; NCT01295736)	Frequent sildenafil use for 12 weeks significantly improved healing of digital ulcers secondary to systemic sclerosis compared to placebo (Hachulla, Hatron et al., 2016).
Tadalafil	Northwestern University; Eli Lilly and Company, USA	Secondary RP	Not applicable (completed 2014; NCT00822354)	Not reported.
Sildenafil	Federal University of São Paulo, Brazil	Secondary RP	Phase III (completed 2016; NCT01347008)	Significant improvement in digital blood flow and symptoms after 8 weeks of treatment compared to placebo (Andrigueti, Ebbing et al., 2017).
Sildenafil	Grenoble University Hospital, France; Pfizer, USA	Mixed RP	Phase II/III (completed 2016; NCT02050360)	Non‐significant improvement in clinical symptoms compared to placebo, with substantial heterogeneity in patient response (Roustit, Giai et al., 2018).
Sildenafil (topical)	Pontifical Catholic University of Chile, Chile	Secondary RP	Phase I (completed 2016; NCT03027674)	Significant improvement in digital blood flow compared to topical nifedipine cream (Wortsman, Del Barrio‐Díaz et al., 2018).
Sildenafil	Angers University Hospital, France	PAD	Phase III (completed 2017; NCT02832570)	Significant improvement in maximal walking time but no significant effects on pain‐free walking time and oxygenation parameters (Omarjee, Le Pabic et al., 2019).
Sildenafil	Angers University Hospital, France	PAD	Phase II/III (not yet recruiting; NCT02387450)	Not yet recruiting.
Sildenafil	Rennes University Hospital, France	PAD	Phase III (ongoing; NCT03686306)	Ongoing.
*Chronic kidney disease*				
Sildenafil	University of Guadalajara, Mexico	Diabetic nephropathy	Not applicable.	Significant reduction in microalbuminuria compared to placebo (Grover‐Páez, Rivera et al., 2007).
PF‐00489791	Pfizer, USA	Diabetic nephropathy	Phase II (completed 2010; NCT01200394)	Significant reduction in macroalbuminuria compared to placebo (Scheele, Diamond et al., 2016).
Mirodenafil	Asan Medical Center; SK Chemicals, South Korea	CKD	Phase I (completed 2011; NCT01232010)	Not reported.
Sildenafil	University of Alabama at Birmingham, USA	Mixed CKD requiring arteriovenous fistula surgery	Not applicable (completed 2018; NCT02414204)	Not reported.
Tadalafil	New England Research Institutes; NHLBI; Massachusetts General Hospital, USA	CKD and AKI in HF	Phase III (completed 2018; NCT01960153)	Not reported.
*Diabetes mellitus and metabolic syndrome*				
Tadalafil	Sahlgrenska University Hospital, Sweden	Hyperglycaemia	Phase I (terminated 2015; NCT01238224)	Durability of study medications could not be guaranteed after the expiry date.
Sildenafil	University of Roma La Sapienza, Italy	Diabetic cardiomyopathy	Phase IV (completed 2009; NCT00692237)	Significant improvement in cardiac kinetic and metabolic indices compared to placebo (Giannetta, Isidori et al., 2012).
Sildenafil	Vanderbilt University; NHLBI; Pfizer, USA	Metabolic syndrome	Phase I/II (completed 2013; NCT01334554)	Not reported.
Tadalafil	Massachusetts General Hospital, USA	Metabolic syndrome	Phase III (completed 2013; NCT01444651)	No improvement in insulin resistance compared to placebo. Potential favourable effects on β‐cell compensation (Ho, Arora et al., 2014).
Vardenafil	Modena Local Health Unit, Italy	Endothelial dysfunction in T2DM	Phase II (completed 2014; NCT02219646)	Significant improvement in endothelial parameters and hypogonadism in men compared with placebo (Santi, Granata et al., 2016).
Tadalafil	University of Roma La Sapienza, Italy	Metabolic syndrome	Phase IV (completed 2015; NCT02554045)	Not reported.
Tadalafil	University of Guadalajara, Mexico	Metabolic syndrome	Phase IV (completed 2015; NCT02595684)	Not reported.
Sildenafil	Vanderbilt University, USA	Hyperglycaemia	Phase IV (completed 2016; NCT01409993 and NCT02129725)	Significant improvement in insulin sensitivity, endothelial parameters, and urine albuminuria after 3 months of PDE5I therapy. Sub‐study investigating insulin signalling in skeletal muscle not yet reported (Ramirez, Nian et al., 2015).
Sildenafil + leucine ± metformin	NuSirt Biopharma, USA	Metabolic syndrome	Phase II (completed 2018; NCT03364335)	Not reported.
Tadalafil	University of Roma La Sapienza, Italy	Diabetic cardiomyopathy	Phase IV (completed 2019; NCT01803828)	Not reported.
Tadalafil	Göteborg University, Sweden	Insulin resistance in T2DM	Phase II (ongoing; NCT02601989)	Ongoing.
Tadalafil	University Center for Health Science, Mexico	Metabolic syndrome	Phase III (ongoing; NCT03905018)	Ongoing.

*Note.* Mode of drug administration is oral unless otherwise specified.

Abbreviations: AKI, acute kidney injury; CKD, chronic kidney disease; ED, erectile dysfunction; HF, heart failure; HFpEF, heart failure with preserved ejection fraction; HFrEF, heart failure with reduced ejection fraction; IRI, ischaemia reperfusion injury; MI, myocardial infarction; NCRR, National Center for Research Resources; NHLBI, National Heart, Lung, and Blood Institute; PAD, peripheral arterial disease; PDE5I, PDE type 5 inhibitor; PH, pulmonary hypertension; RHF, right heart failure; RHTN, treatment‐resistant hypertension; RP, Raynaud's phenomenon; T2DM, type 2 diabetes mellitus.

### 7.3 Drug interactions

Although concomitant use of PDE5Is with nitrates, sGC stimulators and α‐adrenoreceptor antagonists is contraindicated due to the risk of a potent hypotensive interaction (Webb, Freestone et al., 1999), PDE5Is do not have synergistic effects on BP with other antihypertensive agents, such as ACEIs, ARBs, beta‐blockers, CCBs, or thiazide diuretics (Kloner, Mitchell et al., 2003; Webb, Freestone et al., 1999).

Potent inhibitors of the CYP450 system (particularly CYP3A4), including several antiretroviral protease inhibitors, have been shown to modify the pharmacokinetics of PDE5Is. Whilst coadministration has not been associated with changes in the safety or tolerability of either agent (Muirhead, Wulff et al., 2000), cautious use of a lower PDE5I dose is still recommended for patients receiving agents with CYP450 inhibitory properties (Baxter & Preston, 2019).

Lastly, following several years of uncertainty as to whether PDE5Is were associated with changes in cardiac repolarisation, a well‐designed study in healthy males demonstrated that the small increases in corrected QT interval which may be observed at higher doses of the agents are clinically insignificant (Morganroth, Ilson et al., 2004). In fact, an increasing body of experimental evidence suggests that PDE5Is may exert antiarrhythmic effects on ischaemic myocardium through the modulation of voltage‐gated calcium channels, Na^+^/K^+^‐ATPases and β‐adrenoceptor signalling (Hutchings, Anderson et al., 2018).

### 7.4 Nonarteritic anterior ischaemic optic neuropathy

There is still some uncertainty surrounding the safety profile of PDE5Is. In particular, there have been numerous case reports and case series of nonarteritic anterior ischaemic optic neuropathy (NAION) following use of the agents. NAION, an optic neuropathy precipitated by a decrease in perfusion pressure in the prelaminar region of the optic nerve causing ischaemic infarction and sudden, painless vision loss, is a rare condition, but the leading acquired optic neuropathy in adults over 50 years old, with an annual incidence of 2–10 cases per 100,000 in this population. Several experimental and epidemiological studies have failed to establish a definitive effect of PDE5Is on the ocular circulation or on the risk of NAION (Pomeranz, 2016), which is more likely in the presence of cardiovascular risk factors such as ED. However, important insights into this association have recently been provided by two prospective trials using a case–crossover study design, which controls for the effects of recall bias and participant characteristics by defining the day of NAION onset and the day before as the risk period and matching this with control periods in the preceding weeks. The first, a study of 64 definite or probable cases of NAION, found a twofold increase in the risk of NAION within five half‐lives of PDE5I use, extending to 2 days after sildenafil or vardenafil use and 5 days after tadalafil use (Campbell, Walker et al., 2015). These results were echoed by a second crossover trial which identified 279 men with confirmed NAION and revealed that intermittent exposure to a PDE5I within the preceding month also increased the baseline risk of developing NAION twofold (Flahavan, Li et al., 2017). These findings translate to an annual contribution of approximately three additional NAION cases per 100,000 men 50 years and older with weekly PDE5I use. Consequently, PDE5Is are contraindicated in patients with previous NAION or with a hereditary eye disease.

### 7.5 Persistent pulmonary hypertension of the newborn

Concerns have been raised about the safety profile of PDE5Is in the treatment of fetal growth restriction (FGR). Despite generally promising results in preclinical studies and small clinical trials, in July 2018, the Dutch arm of the international Sildenafil TheRapy In Dismal prognosis Early‐onset fetal growth Restriction (STRIDER) series of RCTs was prematurely terminated after interim data suggested an increased risk of persistent pulmonary hypertension of the newborn (PPHN) and neonatal mortality in the intervention group (Groom, Ganzevoort et al., 2018). In the sildenafil group, 17 cases of PPHN were recorded in 64 babies (27%) compared to three of 58 babies (5%) in the placebo group, in the absence of any survival benefit. In the United Kingdom and the New Zealand and Australia STRIDER trials, there was no observable difference in clinically relevant pathological or histological findings between the sildenafil and placebo groups and no significant amelioration of neonatal survival until term age (Sharp, Cornforth et al., 2019). Although the planned meta‐analysis of individual patient data from these trials is eagerly anticipated, it is uncertain whether PDE5Is will have a therapeutic role in pregnancies complicated by FGR.

## 8 CONCLUSIONS

The proven clinical efficacy and excellent safety profile of PDE5Is in the management of ED and PAH has led to their introduction as over‐the‐counter medications as well as prompting significant research interest in the use of these agents for the enhancement of the actions of cGMP in a range of vascular and non‐vascular conditions. In addition to their acute haemodynamic effects, PDE5Is have demonstrated potent anti‐inflammatory, antioxidant, anti‐proliferative and metabolic properties, largely attributable to modulation of the NO–cGMP signalling pathway. A wide array of experimental data suggests a protective effect of PDE5Is in several major organ systems and therapeutic potential in hypertension, chronic kidney disease and diabetes mellitus. In particular, the recent association of long‐term PDE5I treatment with a reduction in all‐cause mortality merits a broader evaluation of PDE5Is. Ultimately, establishing the optimal use and long‐term safety of these agents in CVD will depend on the implementation of larger RCTs, particularly with other comparative treatments, which may be facilitated by the development of novel, longer acting, and more selective PDE5Is.

### 8.1 Nomenclature of targets and ligands

Key protein targets and ligands in this article are hyperlinked to corresponding entries in http://www.guidetopharmacology.org, the common portal for data from the IUPHAR/BPS Guide to PHARMACOLOGY (Harding et al., 2018), and are permanently archived in the Concise Guide to PHARMACOLOGY 2019/20 (Alexander, et al.,2019 2019).

## CONFLICT OF INTEREST

The authors declare no conflict of interest.
